# *Helicobacter Pylori* Infection in Children

**DOI:** 10.4103/1319-3767.48964

**Published:** 2009-04

**Authors:** Shaman Rajindrajith, Niranga M. Devanarayana, Hithanadura Janaka de Silva

**Affiliations:** Department of Paediatrics, Faculty of Medicine, University of Kelaniya, Ragama - 10110, Sri Lanka; 1 Department of Physiology, Faculty of Medicine, University of Kelaniya, Ragama - 10110, Sri Lanka; 2 Department of Medicine, Faculty of Medicine, University of Kelaniya, Ragama - 10110, Sri Lanka

**Keywords:** Abdominal pain, children, *Helicobacter pylori*, stool antigen test, triple therapy

## Abstract

*Helicobacter pylori* infection is a common problem in pediatric practice, and its acquisition is related with poor socioeconomic conditions. Although the organism is thought to be responsible for many diseases, only a handful of them have a direct causal relationship. At present, only a small number of children with well-defined clinical syndromes are benefited from testing and treatment. The treatment should include at least two antibiotics with a proton pump inhibitor.

In 1983, Robin Warren, a pathologist in Perth, reported the presence of “curved bacterium” in the mucosal layer of the gastric biopsy specimen. Together with Barry Marshall, he subsequently isolated the organism from the gastric biopsy specimens and named it ***Campylobacter pyloridis (C. pylori)***,[1] which was ultimately named as ***Helicobacter pylori (H. pylori)***. Marshall and Warren also noted that ***C. pylori (H. pylori)*** infection was associated with gastric and duodenal ulceration.[2] In recognition with this imperative discovery, they were awarded the Nobel Prize for Medicine in 2005. Their discovery initiated a new interest in previously neglected field of gastric microbiology. In 1994, the National Institute of Health consensus conference in USA declared an association between ***H. pylori*** and peptic ulcer disease.[3] During the same year, ***H. pylori*** was identified as a carcinogen associated with gastric adenocarcinoma[4] and gastric non-Hodgkin lymphoma.[5] An association between ***H. pylori*** and gastric mucosa-associated lymphoid tissue lymphoma (MALToma) was identified in 1991.[6] Therefore, after a decade of the isolation of ***H. pylori***, a previously obscured organism, it took a leap to become the most important microbiological agent in the human upper gastrointestinal tract.

Shortly after the landmark paper from Warren and Marshall, ***H. pylori*** (formerly ***C. pylori)*** was identified for the first time in children undergoing upper gastrointestinal endoscopy and having histological features of chronic active gastritis.[7] This was followed by another study, which described the association of the organism with gastroduodenal inflammation.[8] Consequently, a Canadian group of researches described the presence of the organism in the gastric mucosa of the children with antral gastritis and duodenal ulcers. Moreover, they also demonstrated the healing of duodenal ulcers in two patients following the eradication of the organism.[9] These findings firmly established ***H. pylori*** as an important pathogen in pediatric gastroenterology.

## MICROBIOLOGICAL CHARACTERISTICS OF *H. PYLORI*

***H. pylori*** is a spiral, microaerophilic, gram-negative bacterium with four to six unipolar sheathed flagella.[10] The surface of ***H. pylori*** is coated with 12–15 nm ring-shaped aggregates of urease and heat shock protein.[11] The urease enzyme and the heat shock protein B are located almost exclusively within the cytoplasm in the fresh log-phase cultures of ***H. pylori***. In subcultures, urease and heat shock protein B become associated with the bacterial surface, suggesting bacterial autolysis leading to release of protein and adsorption into the bacterial surface.[12] Some of the lipopolysaccharide of the organism mimics the Lewis blood group antigens in structure.[13] This molecular mimicry also helps in the continued existence of ***H. pylori*** in the unfavorable gastric environment.

### Urease

***H. pylori*** is one of the most powerful urea-splitting organisms. Splitting urea to release nitrogen increases the pH of the gastric antrum. Although not essential, this is one factor that helps it to survive in the hostile gastric antrum. The other important role of urease is to provide nitrogen for the protein synthesis by the organism.[14]

### Vacuolating cytotoxin

This protein is capable of inducing vacuolation in eucaryotic cells. The gene for vacuolating cytotoxin (VacA) protein has also been cloned. All Helicobacter strains have VacA gene but only about 50% of them produce VacA protein. It is believed that VacA inserted into the membranes of endosomal vesicles form pores with chloride channel activity. This alters the composition of the anions within the endosomes and subsequently leads to osmotic swelling, which may have effects on the gastric epithelium.[15] Furthermore, it also induces apoptosis, leading to cell death.[16,17] Animal studies have shown that the supernatant from ***H. pylori*** expressing the toxin causes severe acute superficial mucosal injury.[14]

### Cytotoxin-associated antigen

Cytotoxin-associated antigen (CagA) is probably the most important virulent factor in ***H. pylori***. The CagA gene is found in the Cag pathogenic island (PAI), a genome segment of 40 kb that encodes approximately 30 genes.[18] CagA PAI genes are thought to encode a complex syringe-like structure called type IV secretary complex that helps in translocating the CagA protein into the gastric epithelial cells.[19–21] After translocation, CagA is phosphorylated, possibly by known oncogenes[22] and causes rearrangement of the host cytoskeleton and alters cell signaling[18] and perturbs cell cycle control,[23] which are important pathogenic mechanisms of gastroduodenal disease. Both CagA and the secretory system, by independent mechanisms, activate proinflammatory signals and interleukin-8 secretion.[24,25] Furthermore, the CagA PAI-positive strains are known to induce the expression of a DNA-editing enzyme, which leads to accumulation of mutations in the tumor suppressor p53.[26] On the other hand, it had been shown that there are many CagA PAI-negative strains in gastroduodenal ulcers and gastric carcinomas.[14] Therefore, it is possible that other factors such as host immune mechanisms and environmental factors also play a role in the pathogenesis of the ***H. pylori***-associated gastroduodenal disease.

## EPIDEMIOLOGY OF *H. PYLORI*

More than a half of the world's population is infected with ***H. pylori***, which is acquired almost always within the first 5 years of life.[27] In the developed world, the prevalence rates vary from 1.2% to 12.2%.[28–31] In developing countries, the prevalence rates are much higher. The serological prevalence rates of ***H. pylori*** were 15% and 46% in Gambian children aged less than 20 months and 40–60 months, respectively[32] and 45% among Indian children.[33] In Bolivia and Alaska, at the age of 9 years, the seroprevalence was 70% and 69%, respectively.[34] The seroprevalence in preschoolers in Brazil was found to be 69.7%.[35] An age-related increase of the prevalence of ***H. pylori***, irrespective of the economic state of the country, was observed by several independent studies across the world.[29–33,36]

Factors predisposing to ***H. pylori*** infection in children have been studied in detail. The main predisposing factors identified in these studies were low socioeconomic status.[29,37] ***H. pylori*** infection is greater among those living in crowded dwellings.[30] In their study, McCallion ***et al.*** showed that the association between social class and ***H. pylori*** becomes insignificant after adjustment for household density and bed-sharing between a child and an adult. This finding suggests that with regard to the acquisition of ***H. pylori*** infection, social class was acting as a proxy measure for conditions and practices within the household that increase the transmission of the organism from infected to uninfected subjects.[38] Improvements in the standards of living have resulted in a marked reduction in ***H. pylori*** transmission.[39]

## TRANSMISSION OF *H. PYLORI* INFECTION

However hostile, the human stomach is the only identified reservoir for ***H. pylori***. Although extensively studied, efforts to confirm the exact route of transmission have been disappointing. It has been speculated that the person-to-person spread currently appeared to be the most likely mode of transmission, especially between family members.[40,41] Hence, the possible routes are feco–oral, oral–oral and gastro–oral.[42] Thomas ***et al.*** were able to isolate ***H. pylori*** DNA and culture ***H. pylori*** from human feces, suggesting a feco–oral transmission.[43] Oral–oral transmission has also been recognized because ***H. pylori*** was isolated from dental plaques and saliva.[44] Furthermore, gastric–oral transmission was also suggested after the organism was cultured from vomitus.[45,46] This was further strengthened by Perry ***et al.***, who found that this transmission correlates with exposure to an infected family member with gastroenteritis, especially with vomiting.[47] Other possible ways of transmission include water-borne transmission[48] and vector-borne transmission.[49,50] Therefore, it is evident that the current knowledge on the exact modes of transmission is not optimal, and further studies are needed to clarify this clinically and epidemiologically important aspect of ***H. pylori***.

## DIAGNOSIS OF *H. PYLORI* INFECTION

Diagnosis of ***H. pylori*** may be divided into invasive and noninvasive techniques. Invasive tests need an upper gastrointestinal endoscopy (UGIE) and biopsy material for tests, and the noninvasive techniques use other methods. Generally, biopsy cannot be justified, especially in children, unless one wishes to isolate the organism for antibiotic sensitivity testing or there is a clear clinical indication for UGIE. If one opted to test for ***H. pylori*** by biopsies with UGIE, it needs a specimen from multiple regions of the stomach, including antrum, body and transitional zones (i.e., cardia and incisura).

### Invasive testing using biopsies from UGIE

#### Histology

The standard hematoxylin and eosin stain demonstrates inflammation (gastritis type) and ***H. pylori*** itself. Special stains are used to detect ***H. pylori*** if they are lesser in number. Silver stain detects organisms in small numbers but the disadvantage is that it is not possible to study the histology with silver stain and it is expensive. Less-expensive modified Giemsa stain is sensitive and specific for the organism and is much easier to perform than the silver stain.[51]

#### Culture

Culturing this fastidious organism is relatively difficult, expensive and needs special media. The organism is identified as ***H. pylori*** if it is positive for urease, catalase and oxidase and produces a negative reaction for hippurate hydrolysis and nitrate reduction.[51] Currently, cultures are only obtained in research settings and suspected drug resistance.[52]

#### Rapid urease test

The ability of the organism to split urea using the enzyme urease is used to identify ***H. pylori*** in tissue biopsy specimens. A specimen is placed on a commercially available urea-containing medium. The hydrolysis of the urea by urease brings about a color change in the pH-sensitive chemicals in the medium allowing the investigator to identify the presence of the organism. The accuracy of the test is dependent on the number of tissue specimens tested, the location from which the biopsy is obtained, the bacterial load and previous use of antibiotics and proton pump inhibitors as well as the prevalence of the infection in the community.[51]

### Noninvasive tests for *H. pylori*

#### Serology

As in any other infection, ***H. pylori*** infection of the stomach exerts an immunological response in the body. The antibody response in children is to low-molecular weight antigens in the 15–30 kD range and take up to 60 days to develop.[51] Tests of serum immunoglobulin (Ig) A or IgM antibodies are unreliable to detect gastric colonization and, therefore, only IgG antibodies are used in clinical laboratory practice, which is measured using an enzyme-linked immunosorbent assay (ELISA). The presence of IgG antibodies against ***H. pylori*** denotes active infection because once an individual is colonized, the infection continues throughout life unless a course of appropriate eradication therapy is instituted.[53]

Nonetheless, the sensitivity of serological assays is poor in children unless used in the populations in which they were initially developed.[54] The choice of the antigen used in this test to detect the antibody is critical. The strains in Asia are different from those that are circulating in the rest of the world.[55] The sensitivity, specificity and positive and negative predictive values of the same test kit may differ in different ethnic or geographic populations.[56] It is observed that commercially available serum-based immunoassays have a range of sensitivity from 63% to 78%.[57] Besides, the mean antibody levels in young children are significantly lower than in older children and adults and these age-related standard values have not been established for children. Furthermore, most commercial kits have not been validated for children and the cut-off point for adults may be too high for children, resulting in a low sensitivity for the test.[58] Lastly, the antibodies persist even for months after eradication of the infection, reflecting a “scar” of a past infection, rather than indication of an acute infection. Owing to all these problems, the European and North American societies of Pediatric Gastroenterology, Hepatology and Nutrition do not recommend using serological tests in clinical settings until a further validation of serologic commercial kits.[52,55]

#### Salivary IgG antibodies

Commercial kits are available to detect IgG antibodies in the saliva of infected children. Apart from all the disadvantages of the antibody assay discussed above, this test is less sensitive than the urea breath test (UBT).[59] The only advantage of this test is that it is less invasive and can be used in epidemiological studies.

#### UBT

The ^13^C UBT has been used to diagnose ***H. pylori*** infection in children. Isotope urea (^13^C) ingested by the patient is split into ^13^CO_2_ and ammonia. Labeled CO_2_ is absorbed into the bloodstream and is detected and measured in expired air using a mass spectrometer. The sensitivity, specificity, positive predictive value and negative predictive value of UBT were approximately 96%, 93–97%, 91–97% and 97%, respectively in children and adolescents.[60,61] However, in the consensus statement of the European ***Helicobacter pylori*** Study Group, Drumm ***et al.*** state the high sensitivity and specificity of UBT in children over 6 years. Experience in children 5 years and below, particularly in infants, is relatively limited and needs further validation.[55]

#### Stool antigen test

Another novel approach in the diagnosis of ***H. pylori*** infection is the detection of bacterial antigen in stools: ***H. pylori*** stool antigen test. The initially developed stool antigen test was a polyclonal antibody test (Premier Platinum HpSA test; Meridian Diagnostic Inc., Cincinnati, OH, USA) and was found to have variable sensitivities and specificities for the diagnosis of ***H. pylori*** infection. The development of a new ELISA test using monoclonal antibodies (Femtolab ***H. pylori***; Connex, Martinsried, Germany) gave a new dimension and greater precision for stool antigen testing. In comparative studies, stool antigen test using monoclonal antibodies showed a higher sensitivity than the polyclonal test.[62] This test has demonstrated sensitivities, specificities and positive and negative predictive values of 98%, 99%, 98% and 99%, respectively[63] proving that monoclonal stool antigen enzyme immune assay is an excellent tool in diagnosing ***H. pylori*** infection and confirming eradication in children. Therefore, the stool antigen test has now been adopted as part of the standard of care for children with suspected ***H. pylori*** infection.[64]

## *H. PYLORI*–RELATED PATHOLOGICAL CONDITIONS

### Gastritis

Many studies convincingly demonstrate that ***H. pylori*** colonization of the gastric mucosa is associated with gastritis, with chronic inflammatory cell infiltrate in children. The macroscopic nodular gastritis was significantly associated with active chronic gastritis and follicular gastritis.[65,66] Microscopically, the gastric antrum shows lymphonodular hyperplasia and a small number of neutrophils as compared with adults.[67] Colonization of the gastric antrum by ***H. pylori*** is graded as mild, moderate or marked. In children, the number of bacteria present on the gastric mucosa is usually less than that in adults.[51]

### Peptic ulcer disease

There is a wide body of evidence indicating that ***H. pylori*** causes both gastric and duodenal ulceration in children. It had also been compellingly shown that children with duodenal ulcer disease experience less relapse if ***H. pylori*** is eradicated from the gastroduodenal mucosa.[68,69]

### Gastric malignancies

Epidemiological evidence indicates that there is a link between ***H. pylori*** infection and gastric adenocarcinoma and non-Hodgkin lymphoma. However, there are no studies to prove that ***H. pylori*** eradication during childhood prevents subsequent development of gastric malignancies.[52]

### MALToma

The association of ***H. pylori*** and MALToma is an established fact. Therefore, it is recommended to test and treat for ***H. pylori*** in circumstances of histopathological evidence of MALTomas in children.[52]

### Gastro-esophageal reflux disease

Yet another highly controversial topic is the role of ***H. pylori*** in the pathogenesis of gastro-esophageal reflux disease (GORD). Some studies suggest that ***H. pylori*** protects human subjects from developing GORD,[70] whereas others postulate a causative association between them. Some studies have identified an association between the CagA-positive strains and the increased acid secretion that in turn leads to gastro-esophageal reflux.[71] The causative association between ***H. pylori*** and GORD needs further research for confirmation.

### Iron deficiency anemia

Several studies have shown a relationship between ***H. pylori*** and iron deficiency anemia.[72,73] Whether this is caused by the increased iron loss or the decreased iron absorption is not clear yet. According to the First World Congress of Pediatric Gastroenterology, Hepatology and Nutrition working group report, children infected with ***H. pylori*** have lower body iron stores in comparison to age-matched controls.[74]

### Growth faltering in children

Several studies have shown that ***H. pylori*** infection in childhood is associated with growth faltering.[75–78] However, these studies are confounded by the coexistence of variables such as poor socioeconomic status, which may contribute to both the development of malnutrition and the early ***H. pylori*** colonization. Therefore, ***H. pylori*** and growth faltering may be mere associations rather than cause and effect.

### Recurrent abdominal pain

After 20 years of isolating ***H. pylori***, its association and cause of recurrent abdominal pain has been one of the most debated controversies in pediatric gastroenterology. Researchers across the globe who investigated this association have found contradicting results. In these researches, several methods were used to define positive ***H. pylori*** status, including serology, histology, bacterial cultures, rapid urease test and UBT. Some investigators used treatment trials to eradicate ***H. pylori.*** The heterogenicity of their definition of recurrent abdominal pain and the methodologies may have led to the controversies in the results and conclusions.

Many investigators from different parts of the world have observed an association between ***H. pylori*** and recurrent abdominal pain,[79–81] whereas many others have failed to find an association between the same. [82–87] Finally, a meta-analysis of 45 studies in children with ***H. pylori*** and outcome found a weak and inconsistent evidence of an association between ***H. pylori*** infection and classic recurrent abdominal pain.[88] The medical positional statement of the North American Society of Pediatric Gastroenterology, Hepatology and Nutrition regarding ***H. pylori*** infection in children also found no convincing data to support the routine testing of children with recurrent abdominal pain for ***H. pylori.***[52]

## MANAGEMENT OF CHILDREN WITH *H. PYLORI* INFECTION

### When to test children for *H. pylori* infection?

It is important to keep in mind that testing children for Helicobacter infection should be performed only after a reasonable clinical diagnosis. The tests that the clinician selects need to have the ability to diagnose active infection, such as demonstration of the organism by histology or culture from endoscopic biopsy, UBT or fecal antigen test. The following are the current indications for testing:

1. Endoscopically proven gastric or duodenal ulcer
2. Radiographically defined duodenal ulcer
3. Histological evidence of gastric MALToma
4. Pathologically proven gastric metaplasia or atrophic gastritis

It is tempting to test children with recurrent abdominal pain for ***H. pylori***. But, as discussed earlier, there are no convincing data to suggest that the syndrome of recurrent abdominal pain is caused by infection with ***H. pylori***. Therefore, currently, it is not recommended to test these children for ***H. pylori***. Similarly, there are no compelling data to support routinely testing children without symptoms, including those residing in long-term care facilities, children with short stature and those with an increased risk of acquisition of the infection.[52]

### Indications for treatment

1. Documented gastric or duodenal ulcer
2. Histologically proven gastric metaplasia
3. Gastric MALToma
4. Prior documented gastric or duodenal ulcer with current active infection

Yet again, treatment is not indicated in children with recurrent abdominal pain and nonulcer dyspepsia. Furthermore, it is not justifiable to treat children with unexplained short stature or at risk of infection, including asymptomatic children who have a family member with either peptic ulcer or gastric cancer with anti-Helicobacter therapy.[52]

As in any other infection, the treatment is intended to eradiate ***H. pylori*** from the gastroduodenal mucosa. It is vital to accomplish complete eradication as re-infection and development of drug resistance are two important problems in children. Therefore, it is imperative that when treatment is offered, complete courses are given, appropriate protocols that account for common local resistance patterns are followed and treatment is reserved for those patients who will benefit from eradication.[89]

Anti-Helicobacter treatment in children is not effectively evidence-based as that in adults. Most are open-label, case series and uncontrolled anecdotal observations that do not meet the minimum criteria for determining efficacy.[52] There are several reasons why good treatment trials are lacking in the pediatric population. Firstly, prevalence of infection in developed countries is low, and large series could only be collected by multicenter studies. Secondly, such studies are expensive and pharmaceutical companies are not willing to invest money in the pediatric market. Thirdly, in less-developed countries, where prevalence of infection in children is high, facilities to perform clinical trials are not widely available.[90] Therefore, current treatment strategies to eradicate ***H. pylori*** have been developed primarily by using data from adults.

Monotherapy with a single antibiotic has shown very low eradication rates and hence is not recommended as an effective therapy. Several combinations of dual therapies were used by researchers worldwide. In a recent meta-analysis using the metaregression model, Khurana ***et al.*** showed that the combined treatment of amoxicillin and a proton pump inhibitor has a very low eradication rate [e.g., Spain (21%), Mexico (48%), Germany (52%) and Japan (70%)]. However, the efficacy of dual therapy with amoxicillin and nitroimidazole was far more encouraging, with a clearance rate of 84% in European studies when given for 2–6 weeks.[91]

Triple therapy is considered to be the standard treatment for children. Proton pump inhibitor combined with two antibiotics has been shown to be very effective in clearing ***H. pylori*** from the stomach. Current recommendation is treatment with amoxicillin, clarithromycin and a proton pump inhibitor for 2 weeks [Table 1]. An additional two triple therapy regimens that are effective in children include a proton pump inhibitor combined with clarithromycin and metronidazole or amoxicillin and metronidazole combined with bismuth given for a 2-week duration[91] [Table 1].

**Table 1:** Recommended first-line treatment for *Helicobacter pylori* infection in children

Option	Drug	Dosage	Duration
1	Amoxicillin	50 mg/kg/day up to 1 g twice daily	
	Clarithromycin	15 mg/kg/day up to 500 mg twice daily	14 days
	Proton pump inhibitor (e.g., Omeprazole)	1 mg/kg/day up to 20 mg twice daily	
2	Amoxicillin	50 mg/kg/day up to 1 g twice daily	
	Metronidazole	20 mg/kg/day up to 500 mg twice daily	14 days
	Proton pump inhibitor	1 mg/kg/day up to 20 mg twice daily	
3	Clarithromycin	15 mg/kg/day up to 500 mg twice daily	
	Metronidazole	20 mg/kg/day up to 500 mg twice daily	14 days
	Proton pump inhibitor	1 mg/kg/day up to 20 mg twice daily	

In adults, triple therapy based on amoxicillin, clarithromycin and a proton pump inhibitor was shown to be more effective when given for 14 days rather than for 7 days[92], but showed an equal efficacy in 1- and 2-week regimens for children.[91] However, head-to-head comparisons are needed to confirm whether differences exist between these regimens before definite recommendations. Efficacy of the 10-day treatment with proton pump inhibitor-based therapy or bismuth-based therapy is not very widely studied in children. Two studies on 10-day triple therapy and 10-day sequential therapy showed eradication rates of 92%[93] and 97.3%,[94] respectively. However, data regarding efficacy of the 10-day therapy is still inadequate, and therefore, it is not recommended in a recent meta-analysis[91] and a systematic review.[90] Confirmation of ***H. pylori*** eradication should be performed at least 4 weeks after treatment using a noninvasive test such as UBT or stool antigen test.[92]

### Other therapies

Bismuth-based quadruple therapy is considered as the preferred second-line therapy in adults. There is one trial in children using bismuth-based quadruple therapy that showed an eradication rate of 84%.[93] In children, quadruple therapy with metronidazole, clarithromycin, amoxicillin and omeprazole was effective, with an eradication rate of 94%.[95] Furazolidone, metronidazole, amoxicillin and a proton pump inhibitor cured 80% of the cases.[96] Amoxicillin, omeprazole, clarythromycin and Lactobacillus eradicated more than 90% of the infection.[97] Because quadruple therapy is used as a second-line treatment, more pediatric research in this area is urgently needed.

Only one trial has tested the efficacy of sequential therapy in children, which reported eradication of ***H. pylori*** infection in more than 90% of the children.[94] Therapy with a combination of rifabutin, rifamycin or levofloxacin is yet to be tested in children, but it is recommended that these therapeutic options should be based on antimicrobial susceptibility testing.[92]

Recent studies have assessed the value of probiotics in the management of ***H. pylori*** infection. Some studies have shown a higher eradication of infection when probiotics were used in combination with triple therapy in adults.[98] Several studies have shown a significant reduction in the antibiotic-associated side effects and an increase in the overall treatment tolerability[99,100] while some others have failed to show such effects.[98] However, further pediatric studies are needed for a definite identification of the therapeutic value of probiotics in the management of ***H. pylori*** before recommending it as a standard therapy.

## References

[1] Warren JR, Marshall BJ (1983). Unidentified curved bacilli on gastric epithelium in active chronic gastritis. Lancet.

[2] Marshall BJ, Warren JR (1984). Unidentified curved bacilli in the stomach of patient with gastritis and peptic ulceration. Lancet.

[3] (1994). NIH consensus conference. *Helicobacter pylori* in peptic ulcer disease. NIH consensus development panel on *Helicobacter pylori* in peptic ulcer disease. JAMA.

[4] World Health Organization/International Agency of Research on Cancer (1994). Infection with *Helicobacter pylori*. Schistosomes, Liver flukes and *Helicobacter pylori*.

[5] Personnet J, Hansen H, Rodriguez L, Gelb AB, Warnke RA, Jellum E (1994). *Helicobacter pylori* infection and gastric lymphoma. N Engl J Med.

[6] Wotherspoon AC, Oritz-Hidalga C, Falzon MR, Issacson PG (1991). Helicobacter associated gastritis and primary gastric B cell lymphoma. Lancet.

[7] Hill R, Pearman J, Worthy P, Caruso V, Goodwin S, Blincow E (1986). Campylobacter pyloridis and gastritis in children. Lancet.

[8] Cadranel S, Goossens H, De Boeck M, Malengreau A, Rodesch P, Butzler JP (1986). Campylobacter pyloridis in children. Lancet.

[9] Drumm B, Sherman P, Cutz E, Karmail M (1987). Association of Campylobacter pylori on the gastric mucosa with antral gastritis in children. N Engl J Med.

[10] Goodwin CS, McCulloch RK, Armstrong JA, Wee SH (1987). Unusual cellular fatty acid and distinctive ultrastructure in a new spiral bacterium (Campylobacter pyloridis) from the human gastric mucosa. J Med Microbiol.

[11] Austin J, Doig WP, Stewart M, Trust TJ (1992). Structural comparison of urease and a GroEL analog from *Helicobacter pylori*. J Bacteriol.

[12] Phadnis SH, Paelow MH, Levy M, Ilver D, Caulkins CM, Conners JB (1996). Surface localization of *Helicobacter pylori* urease and heat shock protein homolog requires bacterial autolysis. Infec Immun.

[13] Applemelk BJ, Simoons-Smit I, Negrini R, Moran AP, Aspinall GO, Forte JG (1996). Potential role of molecular mimicry between *Helicobacter pylori* lipopolysaccharide and host Lewis blood group antigens in autoimmunity. Infec Immun.

[14] Peterson WL, Graham DY, Feldman M, Friedman, Sleisenger MH, Scharschmidt BF (2002). Helicobacter pylori. Sleisenger and Fordtran's gastrointestinal and liver disease pathophysiology diagnosis management.

[15] Amieva MR, El-Omar EM (2008). Host-bacterial interactions in *Helicobacter pylori* infection. Gastroenterology.

[16] Willhite DC, Blanker SR (2004). *Helicobacter pylori* vaculating cytotoxin enters cells, localizes to mitochondria, and induces mitochondrial membrane permeability changes correlated to toxin channel activity. Cell Microbiol.

[17] Yamasaki E, Wada A, Kumaroti A, Nakagawa I, Funao J, Nakayama M (2006). *Helicobacter pylori* vacuolating cytotoxin induces activation of the proapoptotic proteins Bax and Bak, leading to cytochrome c release and cell death, independent of vaculation. J Biol Chem.

[18] Figueiredo C, Machado JC, Yamaoka Y (2005). Pathogenesis of *Helicobacter pylori*. Helicobacter.

[19] Stein M, Rappuoli R, Covacci A (2000). Tyrosin phosphorylation of the Helicobacter pylori CagA antigen after cag-driven host cell translocation. Proc Natl Acad Sci USA.

[20] Asahi M, Azuma T, Ito S, Ito Y, Suto H, Nagai Y (2000). *Helicobacter pylori* CagA protein can be tyrosin phosphorilated in gastric epithelial cells. J Exp Med.

[21] Odenbreit S, Puls J, Sedlmaier B, Gerland E, Fischer W, Haas R (2000). Translocation of *Helicobacter pylori* CagA into gastric epithelial cells by type IV secretion. Science.

[22] Tammer I, Brandt S, Hartig R, König W, Backert S (2007). Activation of Abl by *Helicobacter pylori*:a novel kinase for CagA and crucial mediator of host cell scattering. Gastroenterology.

[23] Chang YJ, Wu MS, Lin JT, Pestell RG, Blaser MJ, Chen CC (2006). Mechanisms for *Helicobacter pylori* CagA-induced cyclin D1 expression that affect cell cycle. Cell Microbiol.

[24] Shibata W, Hirata Y, Maeda S, Ogura K, Ohmae T, Yanai A (2006). CagA protein secreted by the intact type IV secretion system leads to gastric epithelial inflammation in the Mongolian gerbil model. J Pathol.

[25] Kim SY, Lee YC, Kim HK, Blaser MJ (2006). *Helicobacter pylori* CagA transfection of gastric epithelial cells induces interleukin-8. Cell Microbiol.

[26] Matsumoto Y, Marusawa H, Kinoshita K, Endo Y, Kou T, Morisawa T (2007). *Helicobacter pylori* infection triggers aberrant expression of activation-induced cytidine deaminase in gastric epithelium Nat Med.

[27] Suerbaum S, Michetti P (2002). *Helicobacter pylori* infection. N Engl Med.

[28] Rothenbacher D, Bode G, Berg G, Gommel R, Gonser T, Alder G (1998). Prevalence and determinants of *Helicobacter pylori* infection in preschool children: A population based study from Germany. Int J Epidemiol.

[29] Opekun AR, Gilger MA, Denyes SM, Nirken MH, Philip SP, Osato MS (2000). *Helicobacter pylori* infection in children of Texas. J Pediatr Gastroenterol Nutr.

[30] Wizla-Derambure N, Michaud L, Ategbo S, Vincent P, Ganga-Zandzou S, Turck D (2001). Familial and community environmental risk factors for *Helicobacter pylori* infection in children and adolescents. J Pediatr Gastroenterol Nutr.

[31] Mourad-Baars PE, Verspaget HW, Mertens BJ, Luisa Merain M (2007). Low prevalence of *Helicobacter pylori* infection in young children in the Netherlands. Eur J Gastroenterol Hepatol.

[32] Sullivan PB, Thomas JE, Wight DG, Neale G, Easthan EJ, Corrah T (1990). *Helicobacter pylori* in Gambian children with chronic diarrhoea and malnutrition. Arch Dis Child.

[33] Kate V, Ananthakrishanan N, Ratnakar C, Badrinath S (2001). Anti-H.pylori IgG seroprevalence rates in asymptomatic children and adults form south India. Indian J Med Microbiol.

[34] Gold BJ (2001). *Helicobacter pylori* infection in children. Curr Prob Pediatr.

[35] Rocha GA, Rocha AM, Silva LD, Samtos A, Bocewicz AC, Queiroz RM (2003). Transmission of *Helicobacter pylori* infection in families of preschool-aged children from Minas Gerais, Brazil. Trop Med Int Health.

[36] Malaty HM, Haveman T, Graham DY, Fraley K (2002). *Helicobacter pylori* infection in asymptomatic children: Impact of epidemiological factors on accuracy of diagnostic tests. J Ped Gastroenterol Nutr.

[37] Ndip RN, Malange AE, Akoachere JF, MacKay WG, Titanji VP, Weaver LT (2004). *Helicobacter pylori* antigens in faeces of asymptomatic children in the Buea and Limbe health districts of Cameroon: A pilot study. Trop Med Int Health.

[38] McCallion WA, Murray LJ, Bailie AG, Dalzell AM, O'Reilly OJ, Bamford KB (1996). *Helicobacter pylori* in children: Relation with current household living conditions. Gut.

[39] Tkachenko M, Zhannat NZ, Erman LV, Blashenkova EL, Isachenko SV, Isachenko OB (2007). Dramatic changes in the prevalence of Helicobacter pylori infection during childhood: A 10 year follow-up study in Russia. J Peditr Gastroenterol Nutr.

[40] Weyermann M, Adler G, Brenner H, Rothenbacher D (2006). The mother as source of *Helicobacter pylori* infection. Epidemiology.

[41] Kivi M, Johansson AL, Reilly M, Tindberg Y (2005). *Helicobacter pylori* status in family members as risk factors for infection in children. Epidemiol Infect.

[42] Gold BJ (2001). New approach to *Helicobacter pylori* infection in children. Curr Gastroenterol Rep.

[43] Thomas JE, Gibson GR, Darboe MK, Dale A, Weaver LT (1992). Isolation of *Helicobacter pylori* from human faeces. Lancet.

[44] Goosen C, Theron J, Ntsala M, Maree FF, Olckers A, Botha SJ (2002). Evaluation of a novel heminested PCR assay based on the phosphoglucosamine mutase gene for detection of *Helicobacter pylori* in saliva and dental plaque. J Clin Microbiol.

[45] Leung WK, Siu KL, Kwok CK, Chan S, Sung R, Sung JJ (1999). Isolation of *Helicobacter pylori* from vomitus of children and its implication in gastro-oral transmission. Am J Gastroenterol.

[46] Personnet J, Shmuely H, Haggeerty T (1999). Fecal and oral shedding of *Helicobacter pylori* from healthy infected adults. JAMA.

[47] Perry S, de la Luz Sanchez M, Yang S, Haggerty TD, Hurst P, Perez-Perez G (2006). Gastroenteritis and transmission of *Helicobacter pylori* infection in Households. Emerg Infect Dis.

[48] Klein PD, Graham DY, Gaillour A, Gaillour A, Opekun AR, Smith EO (1991). Water source as risk factor for *Helicobacter pylori* infection in Peruvian children: Gastrointestinal physiology working group. Lancet.

[49] Grubel P, Hoffman JS, Chong FK, Burstein NA, Mepani C, Cave DR (1997). Vector potential of house fly (Musca domastica) for *Helicobacter pylori*. J Clin Microbiol.

[50] Grubel P, Haung L, Masubuchi N, Stuzenberger, Cave DR (1998). Detection of *Helicobacter pylori* DNA in house fly (Musca domastica). Lancet.

[51] Rowland M, Bourke B, Drumm B, Walker WA, Goulet O, Kleinman RE, Sherman PM, Shneider BL, Sanderson IR (2004). *Helicobacter pylori* and peptic ulcer disease. Pediatric Gastrointestinal disease.

[52] Gold BD, Colletti RB, Abbott M, Czinn S, Elitsur Y, Hassall E (2000). *Helicobacter pylori* infection in children: Recommendation for diagnosis and treatment. J Pediatr Gastroenterol Nutr.

[53] Day AS, Sherman PM (2002). Accuracy of office-based immunoassay for the diagnosis of *Helicobacter pylori* infection in children. Helicobacter.

[54] Makristathis A, Hirschl AM, Lehours P, Megraud F (2002). Diagnosis of *Helicobacter pylori* infection. Helicobacter.

[55] Drumm B, Koletzko S, Oderda G (2000). Europian Paediatric task force on *Helicobacter pylori. Helicobacter pylori* infection in children: A consensus statement. J Pediatr Gastroenterol Nutr.

[56] De Olivaria AM, Rocha GA, Queiroz DM, Mendes EN, de Carvalho AS, Ferrari TC (1999). Evaluation of enzyme-linked immunosorbant assay for the diagnosis of *Helicobacter pylori* infection in children from different age grups with and without duodenal ulcer. J Pediatr Gastroenterol Nutr.

[57] Czinn SJ (1999). Serodiagnosis of *Helicobacter pylori* in Pediatric patients. J Pediatr Gastroenterolo Nutr.

[58] Westblom TU, Madan E, Gudipati S, Midkiff BR, Czinn SJ (1992). Diagnosis of *Helicobacter pylori* infection in adult and pediatric patients by using Pyloriset: A rapid latex agglutination test. J Clin Microbiol.

[59] Bode G, Marchildon P, Peacock J, Brenner H, Rothenbacher D (2002). Diagnosis of *Helicobacter pylori* infection in children: Comparison of a salivary immunoglobulin G antibody test with the 13C urea breath est. Clin Diagn Lab Immunol.

[60] Kawakami E, Machado RS, Reber M, Patricio FR (2002). 13C-Urea breath test with infrared spectroscopy for diagnosing *Helicobacter pylori* infection in children and adolescents. J Pediatr Gastroenterol Nutr.

[61] Megraud F (2005). On behalf of the European Paediatric Task Force on *Helicobacter pylori*. Comparison of non-invasive tests to detect *Helicobacter pylori* infection in children and adolescents: Results of a multicenter European study. J Pediatr.

[62] Makristathis A, Barousch W, Pasching E, Binder C, Kuderna C, Apfalter P (2000). Two enzyme immunoassays and PCR for detection of *Helicobacter pylori* in stool specimens from pediatric patients before and after eradication therapy. J Clin Microbiol.

[63] Koletzko S, Konstantopopulos N, Bosman D, Feydt-Schmidt A, vander Ende A, Kalach N (2003). Evaluation of a novel monoclonal enzyme immunoassay for detection of *Helicobacter pylori* antigen in stool from children. Gut.

[64] Crone J, Gold BD (2004). *Helicobacter pylori* in pediatrics. Helicobacter.

[65] Bahu MG, da Silvaria TR, Maguilnick I, Ulbrich-Kulczynski J (2003). Endoscopic nodular gastritis: An endoscopic indicator of high-grade bacterial colonization and severe gastritis in children with *Helicobacter pylori*. J Pediatr Gastroenterol Nutr.

[66] Uc A, Chong SK (2002). Treatment of *Helicobacter pylori* gastritis improves dyspeptic symptoms in children. J Pediatr Gastroenterol Nutr.

[67] Genta RM, Hammer HW, Graham DY (1993). Gastric lymphoid follicles in *Helicobacter pylori* infection: Frequency, distribution and response to triple therapy. Hum Pathol.

[68] Goggin N, Rowland M, Imrie C, Walsh D, Clyne M, Drumm B (1998). Effect of *Helicobacter pylori* eradication on the natural history of duodenal ulcer disease. Arch Dis Child.

[69] Huang FC, Chang MH, Hsu HY, Lee PI, Shun CT (1999). Long-term follow-up of duodenal ulcer in children before and after eradication of *Helicobacter pylori*. J Pediatr Gastroenterol Nutr.

[70] Blaster MJ (1998). *Helicobacter pylori* and gastric disease. BMJ.

[71] Gold BD (2003). Outcome of pediatric gastroesophageal reflux disease: In the first year of life, in childhood and in adults… oh and should we really leave *Helicobacter pylori* alone. J Pediatr Gastroenterol Nutr.

[72] Choi JW (2003). Does *Helicobacter pylori* infection related to iron deficiency anaemia in prepubescent children under 12 years of age. Acta Paediatr.

[73] Choe YH, Kim SK, Hong YC (2003). The relationship between *Helicobacter pylori* infection and iron deficiency: Seroprevalence study in 937 pubescent children. Arch Dis Child.

[74] Sherman P, Czinn S, Drumm B, Gottrand F, Kawagami E, Madrazo A (2002). *Helicobacter pylori* infection in children and adolescents: Working group report of the first world congress of Pediatric Gastroenterology Hepatology and nutrition. J Pediatr Gastroenterol Nutr.

[75] Fall CH, Goggin PM, Hawtin P, Fine D, Duggleby S (1997). Growth in infancy, infant feeding, childhood living conditions, and *Helicobacter pylori* infection at age 70. Arch Dis Child.

[76] Perri F, Pastore M, Leandro G, Clemente R, Ghoos Y, Peeters M (1997). Helicobacter pylori infection and growth delay in older children. Arch Dis Child.

[77] Thomas JE, Dale A, Bunn JE, Harding M, Coward WA, Cole TJ (2004). Early *Helicobacter pylori* colonization: The association with growth faltering in the Gambia. Arch Dis Child.

[78] Mera RM, Correa P, Fonthan EE, Reina JC, Pradilla A, Alzate A (2006). Effects of a new *Helicobacter pylori* infection on height and weight in Colombian children. Ann Epidemiol.

[79] Frank F, Stricker T, Stallmach T, Braegger CP (2000). *Helicobacter pylori* infection in recurrent abdominal pain. J Pediatr Gastroenterol Nutr.

[80] Kimia A, Zahavi H, Shaprio R, Rosenbach Y, Hirsh A, Drudz T (2000). The role of *Helicobacter pylori* gastritis in children with recurrent abdominal pain. Indian Med Assoc J.

[81] Ozen H, Dinler G, Akyon Y, Kocak N, Yuce A, Gurakan F (2001). *Helicobacter pylori* infection and recurrent abdominal pain in Turkish children. Helicobacter.

[82] Macarthur C, Saunders N, Feldman W, Ipp M, Winder-Lee P, Roberts S (1999). *Helicobacter pylori* and childhood recurrent abdominal pain: Community based case-control study. Br Med J.

[83] Hardikar W, Colin F, Arnold S, Frank O, Keith G (1996). *Helicobacter pylori* and recurrent abdominal pain. J Pediatr Gastroenterol Nutr.

[84] Bode G, Brenner H, Adler G, Rothenbacher D (2003). Recurrent abdominal pain in children: Evidence from a population-based study that social and familial factors play a major role but not *Helicobacter pylori* infection. J Psychosom Res.

[85] Bansal D, Patwari AK, Malhotra VL, Malhotra V, Anand VK (1998). *Helicobacter pylori* infection in recurrent abdominal pain. Indian Pediatr.

[86] Wewer V, Anderson LP, Paerregaard A, Gernow A, Hansen JP, Matzen P (2001). Treatment of *Helicobacter pylori* in children with recurrent abdominal pain. Helicobacter.

[87] Devanarayana NM, de Silva DG, de Silva HJ (2008). Aetiology of recurrent abdominal pain in a cohort of Sri Lankan children. J Paediatr Child Health.

[88] Macarthur C, Saunders N, Feldman W (1995). *Helicobacter pylori*, gastroduodenal disease, and recurrent abdominal pain in children. JAMA.

[89] Campbell DI, Thomas JE (2005). *Helicobacter pylori* infection in paediatric practice. Arch Dis Child Educ Pract.

[90] Oderda G, Rapa A, Bona G (2000). A systematic review of *Helicobacter pylori* eradication treatment schedules in children. Aliment Pharmacol Ther.

[91] Khurana R, Fischbach L, Chiba N, van Zanten SV, Sherman PM, George BA (2007). Meta-analysis: *Helicobacter pylori* eradication treatment efficacy in children. Aliment Pharmacol Ther.

[92] Malfertheiner P, Megaraud F, O'Morain C, Bazzoli F, El-Omar E, Graham D (2007). Current concepts in the management of *Helicobacter pylori* infection: The Maastricht III consensus report. Gut.

[93] Bahremand S, Nematollahi LR, Fourutan H, Tirgari F, Nouripour S, Mir E (2006). Evaluation of triple and quadruple *Helicobacter pylori* eradication therapies in Iranian children: A randomized clinical trial. Eur J Gastroenterol Hepatol.

[94] Francavilla R, Lionetti E, Castellaneta SP, Magista AM, Boscarelli G, Piscitelli D (2005). Improved efficacy of 10-day sequential treatment for *Helicobacter pylori* in children: A randomized trial. Gastroenterology.

[95] Chan KL, Zhou H, Ng DK, Tam PK (2001). A prospective study of one-week non-bismuth quadruple therapy for childhood *Helicobacter pylori* infection. J Pediatr Surg.

[96] Carvalho AS, Queiroz DM, Maides EN, Rocha GA (1998). Triple antimicrobial therapy plus H2-receptor antagonists or omeprazole in the eradication of *Helicobacter pylori* in children with duodenal ulcer. Gut.

[97] Sykora J, Valeckova K, Amlerova J, Siala K, Dedek P, Watkins S (2005). Effects of specially designed fermented milk product containing probiotic Lactobacillus casei DN-114 001 and the eradication of *H.pylori* in children: A prospective randomized double-blind study. J Clin Gastroenterol.

[98] Kim MM, Kim N, Lee SH, Park YS, Hwang JH, Kim JW (2008). The effects of probiotics on PPI triple therapy for *Helicobacter pylori* eradication. Helicobacter.

[99] Lionetti E, Miniello VL, Casterllaneta SP, Magista AM, de Canio A, Maurogiovanni G (2006). Lactobacillus reuteri therapy to reduce side-effects during anti-*Helicobacter pylori* treatment in children: A randomized, placebo controlled trail. Aliment Pharmacol Ther.

[100] Armuzzi A, Cremonini F, Bartolozzi F, Conducci F, Candelli M, Ojetti V (2001). The effects of oral administration of Lactobacillus GG on antibiotic-associated gastrointestinal side-effects during *Helicobacter pylori* eradication therapy. Aliment Pharmacol Ther.

